# Supplementary material for: Concurrent human TMS-EEG-fMRI enables monitoring of oscillatory brain state-dependent gating of cortico-subcortical network activity
Source: Commun Biol. 2020 Jan 22;3:40. doi: 10.1038/s42003-020-0764-0 (PMC6976670; doi:10.1038/s42003-020-0764-0)
Supplement: Supplementary file 1 — Supplemental Information [file 42003_2020_764_MOESM1_ESM.docx]

**Supplementary Materials: Supplementary Figures and Table**


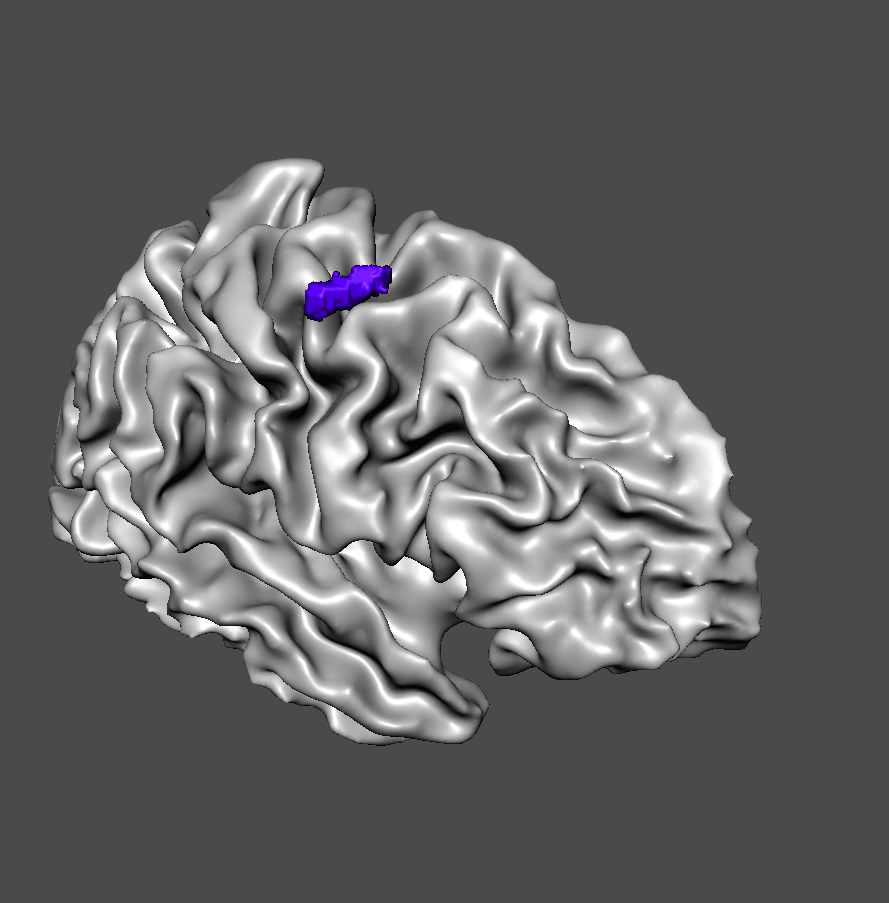

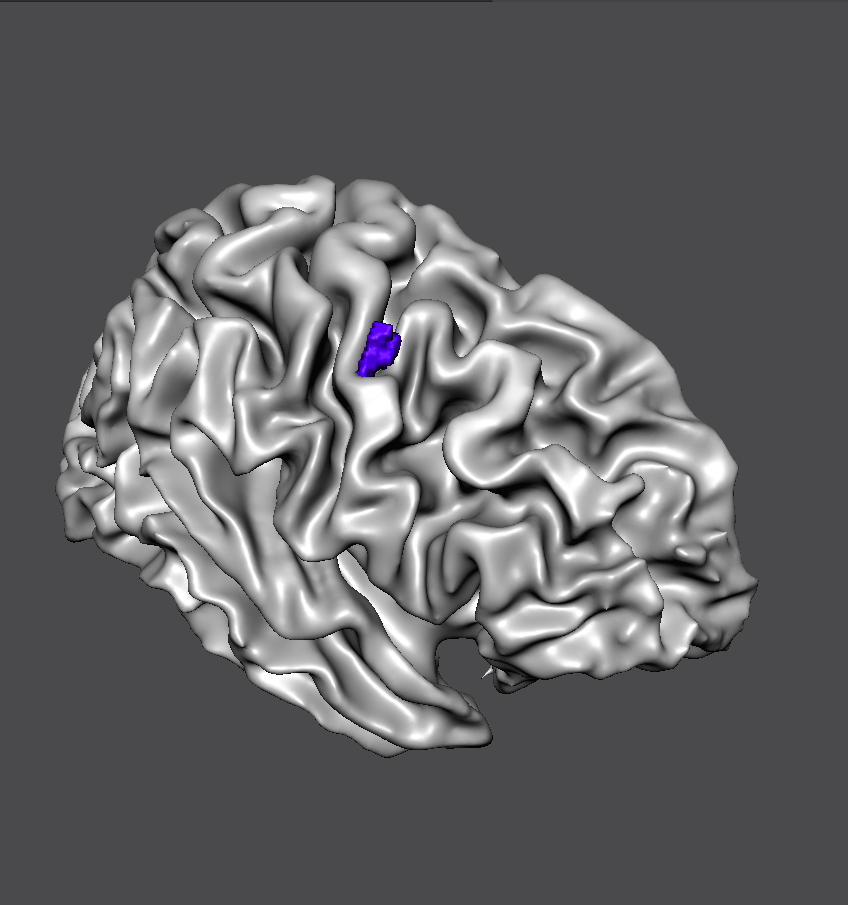


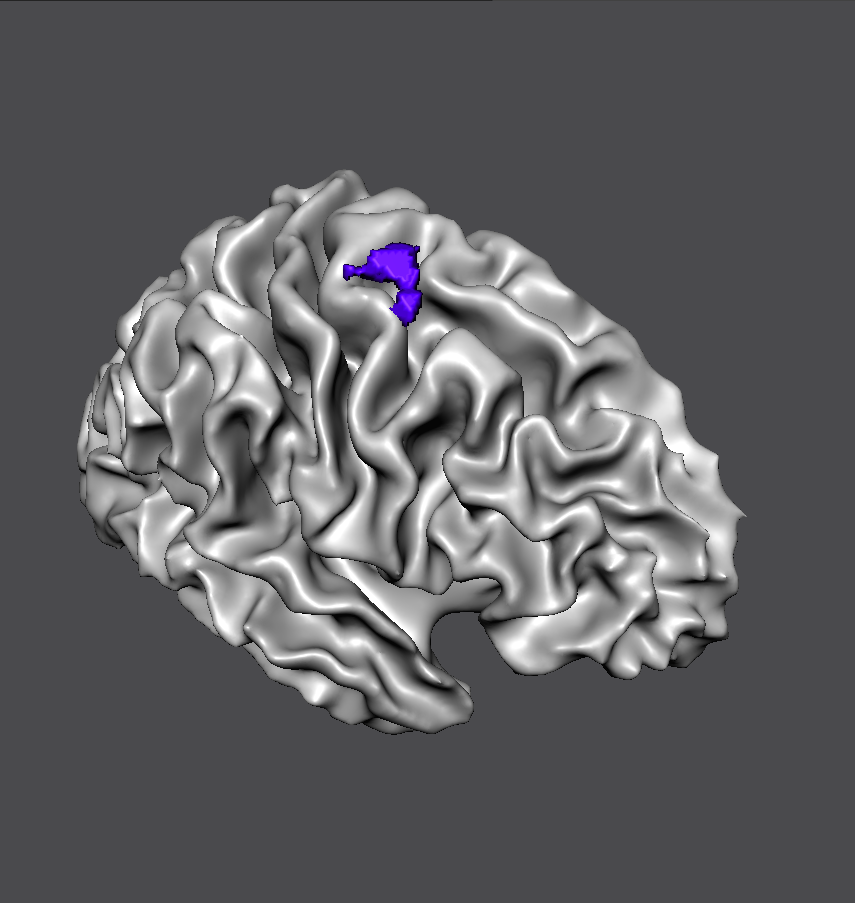

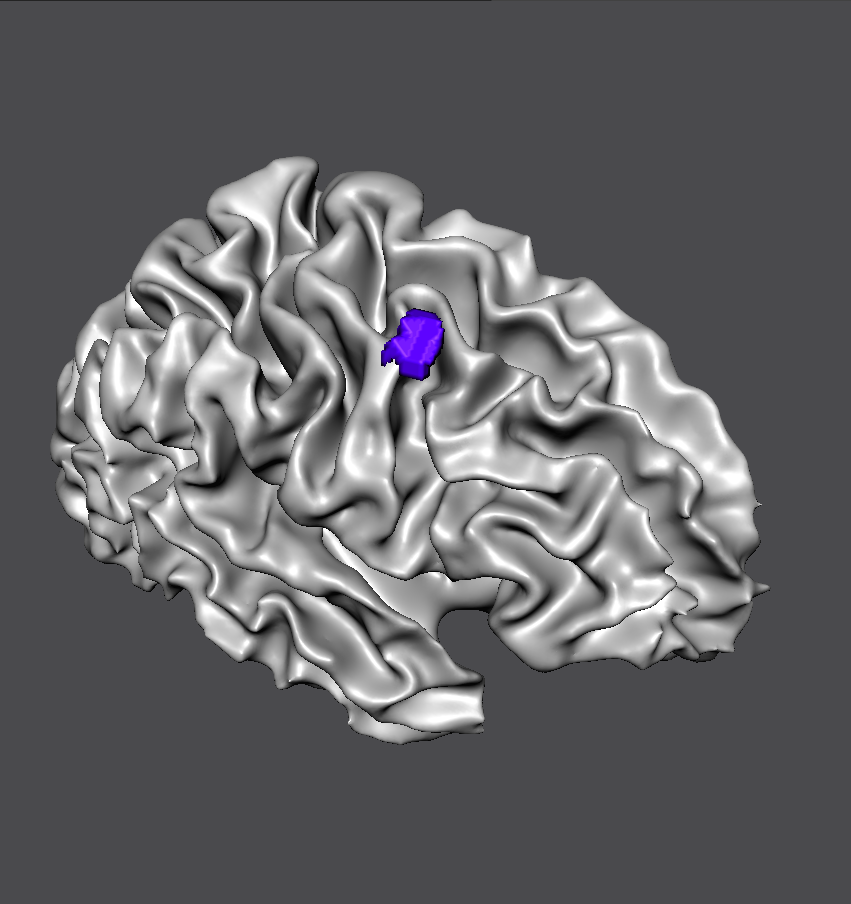


**Supplementary Fig.1. TMS target sites**

TMS target site per participant as defined based on the independent finger tapping localizer fMRI session. Target region is superimposed on the right hemisphere’s cortex mesh reconstruction of the corresponding subject.

**
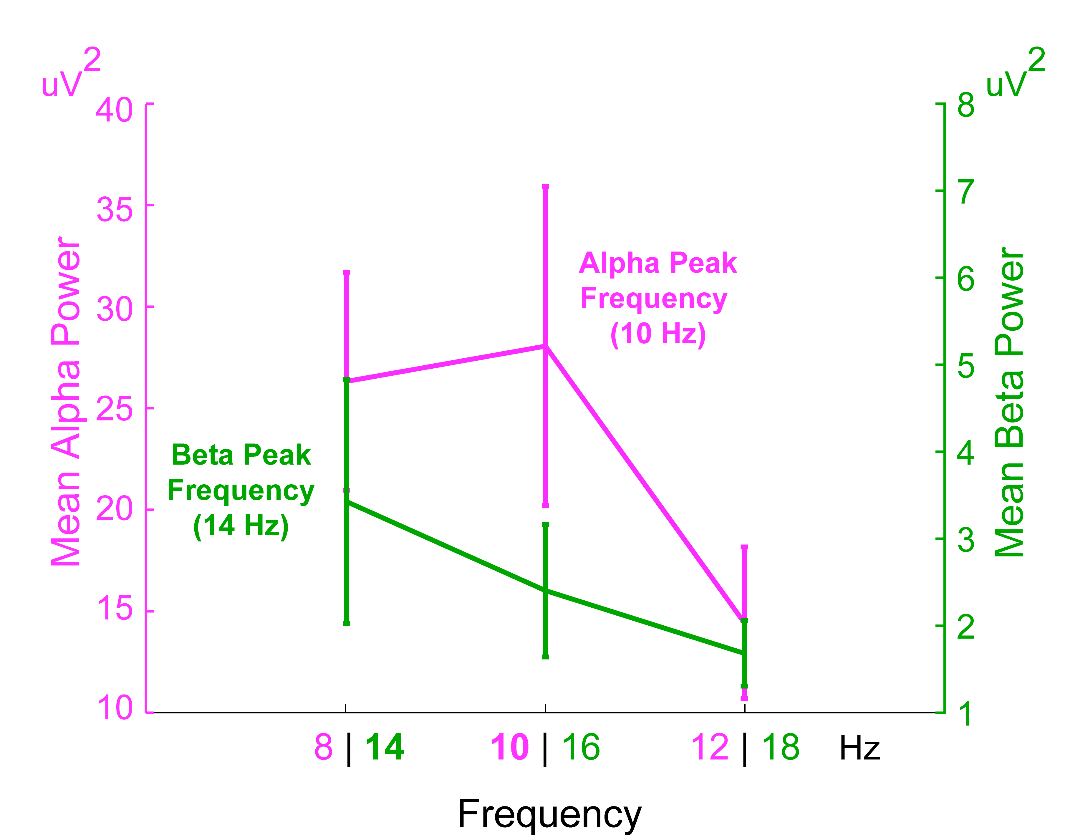
**

**Supplementary Fig.2. Mean power in the alpha and beta range**

Mean power spectra at the Alpha site (8-12 Hz alpha range; pink line) and Motor Beta site (14-18 Hz low-beta range; green line). The alpha peak frequency is 10 Hz, whereas the low-beta power peaked at a frequency of 14 Hz. Error bars represent standard error of the mean (SEM).

**
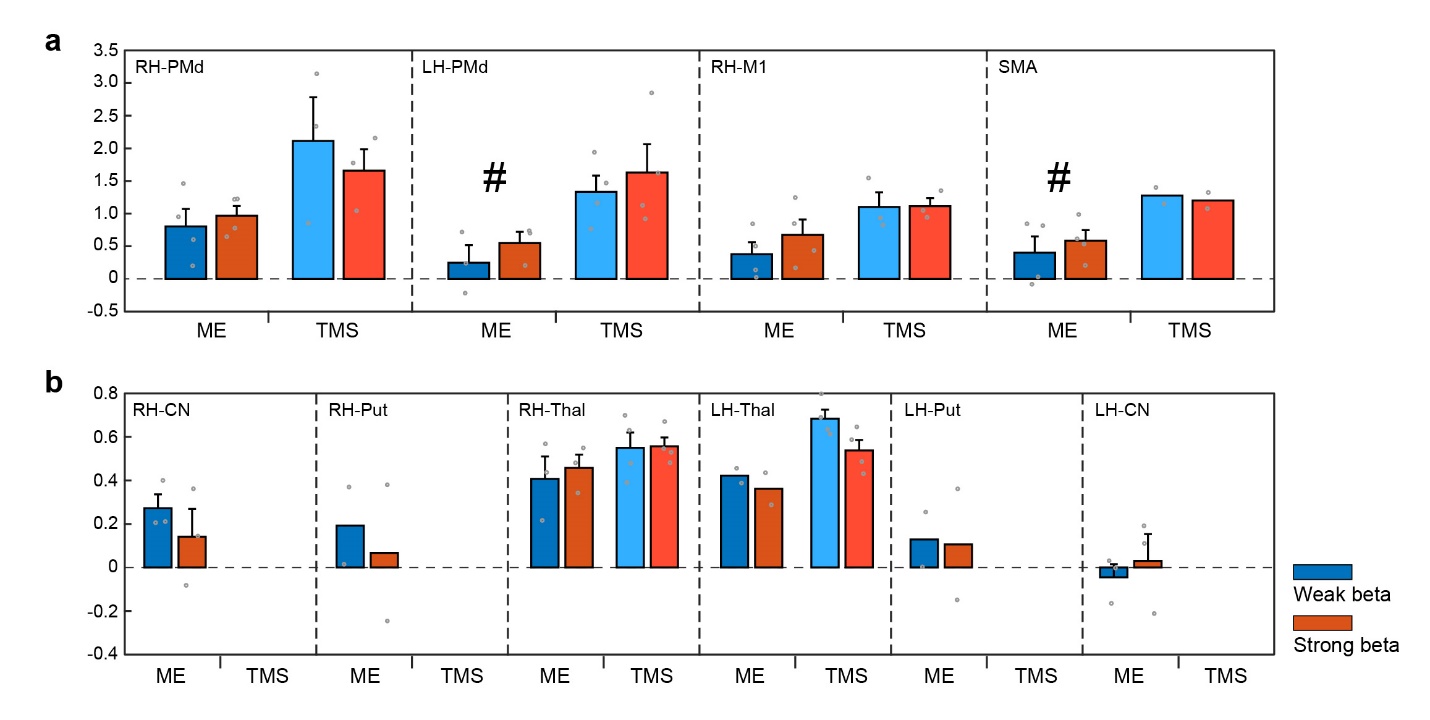
**

**Supplementary Fig.3.** **Strong Pre-TMS *beta* power does *not* shunt TMS-induced activity**

Group mean (n=4) of TMS-evoked BOLD responses for ‘low’ (blue bars) and ‘high’ (red bars) pre-TMS ***beta*** power trials in **a** cortical ROIs and **b** subcortical ROIs. Each category contains 40 trials (per participant) reflecting beta power fluctuations sampled at the TMS target site. In contrast to alpha power, TMS-evoked fMRI responses are not inversely related to beta power (cf. difference between red and blue bars here to difference in Figure 3 and 4). # indicates significance (p<.05) in at least one of the participants. Only ROIs that were identified in two or more participants are shown. Error bars represent SEM (for ROIs identified in 3 or more participants). Formatting and color-coding are identical to Figure 3, except that circles represent individual participants rather than individual trials.

**
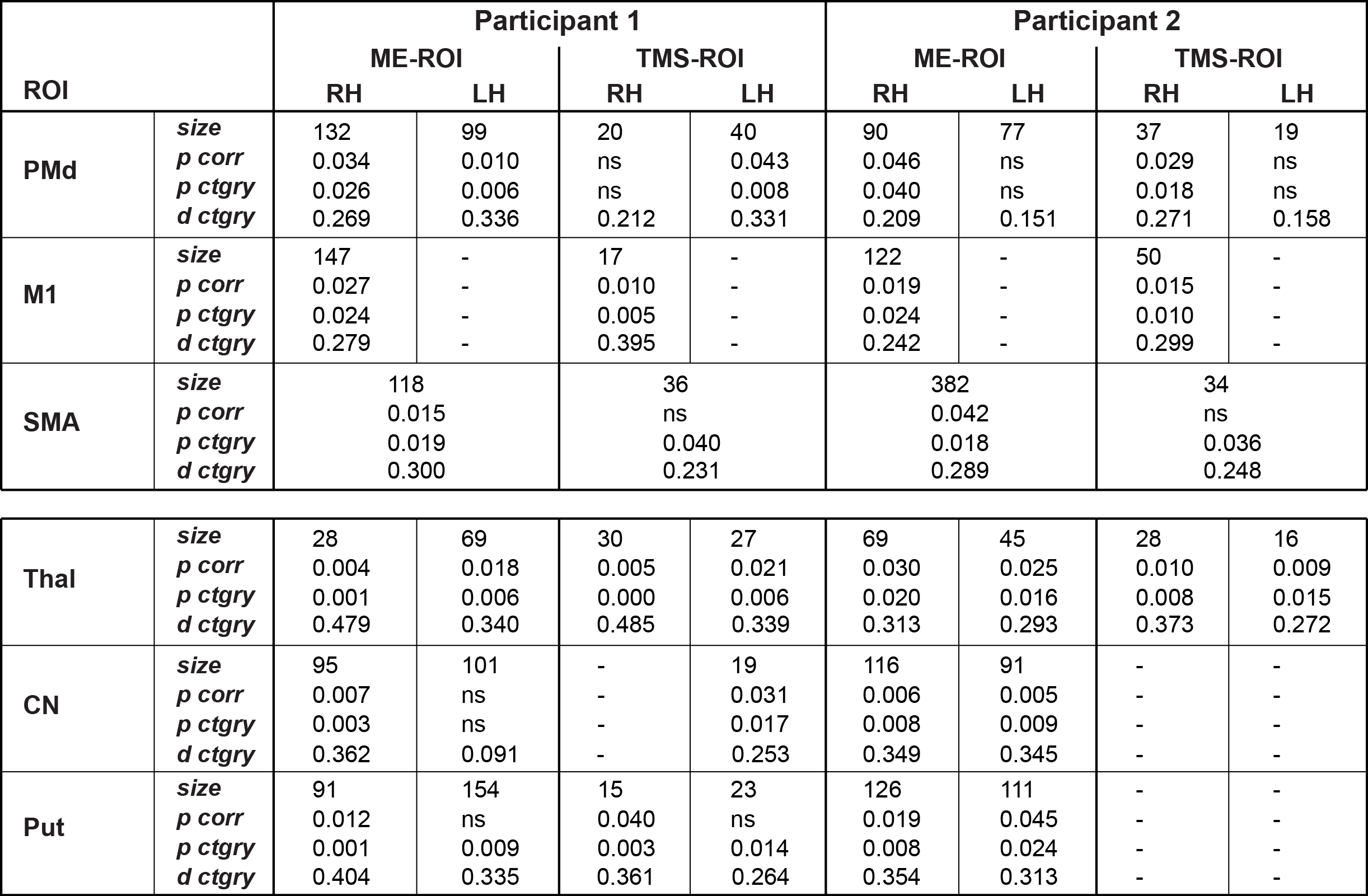
**

**Supplementary Table 1. Overview of the effect of pre-TMS alpha power modulations on TMS-induced fMRI activations across all included ROIs per participant.**

Summary of the size of each ROI (functional voxel count), together with the significance level (*p* value) and effect size (*d*) of the performed correlation (‘corr’) and category (‘ctgry’) analyses for both the ‘motor execution’ and ‘TMS’ ROIs in high activators. All significant effects reflected a *negative* correlation between pre-TMS alpha power and BOLD response (corr) or a *higher* BOLD response in weak compared to strong power trials (ctgry). Effect sizes (*d*; Cohen’s D for dependent samples) indicate the standardized mean change as calculated by dividing the mean difference scores (between the evoked BOLD responses in the two categories) by the standard deviation of these difference scores. a hyphen indicates that the given ROI could not be determined (i.e., no significant activation was observable in the whole-brain analyses at q(FDR) < 0.05). ‘ns’ = non-significant (i.e., p > 0.05); PMd: dorsal PreMotor cortex, M1: Primary Motor Cortex, SMA: Supplementary Motor Area, CN: Caudate Nucleus, Put: Putamen, Thal: Thalamus. RH: right hemisphere, LH: left hemisphere.
