# Supplementary material for: Concurrent human TMS-EEG-fMRI enables monitoring of oscillatory brain state-dependent gating of cortico-subcortical network activity
Source: Commun Biol. 2020 Jan 22;3:40. doi: 10.1038/s42003-020-0764-0 (PMC6976670; doi:10.1038/s42003-020-0764-0)
Supplement: Supplementary file 2 — Reporting Summary [file 42003_2020_764_MOESM2_ESM.pdf]

nature research

Corresponding author(s): J.Peters

Last updated by author(s): Dec 22, 2019

Reporting Summary

Nature Research wishes to improve the reproducibility of the work that we publish. This form provides structure for consistency and transparency in reporting. For further information on Nature Research policies, see Authors & Referees and the Editorial Policy Checklist.

Statistics

For all statistical analyses, confirm that the following items are present in the figure legend, table legend, main text, or Methods section.

n/a	Confirmed	
☐	☒	The exact sample size (*n*) for each experimental group/condition, given as a discrete number and unit of measurement
☐	☒	A statement on whether measurements were taken from distinct samples or whether the same sample was measured repeatedly
☐	☒	The statistical test(s) used AND whether they are one- or two-sided<br>*Only common tests should be described solely by name; describe more complex techniques in the Methods section.*
☐	☒	A description of all covariates tested
☐	☒	A description of any assumptions or corrections, such as tests of normality and adjustment for multiple comparisons
☐	☒	A full description of the statistical parameters including central tendency (e.g. means) or other basic estimates (e.g. regression coefficient) AND variation (e.g. standard deviation) or associated estimates of uncertainty (e.g. confidence intervals)
☐	☒	For null hypothesis testing, the test statistic (e.g. *F*, *t*, *r*) with confidence intervals, effect sizes, degrees of freedom and *P* value noted<br>*Give P values as exact values whenever suitable.*
☒	☐	For Bayesian analysis, information on the choice of priors and Markov chain Monte Carlo settings
☒	☐	For hierarchical and complex designs, identification of the appropriate level for tests and full reporting of outcomes
☐	☒	Estimates of effect sizes (e.g. Cohen's *d*, Pearson's *r*), indicating how they were calculated

Our web collection on statistics for biologists contains articles on many of the points above.

Software and code

Policy information about availability of computer code

Data collection	Stimulus presentation: Presentation 14 (www.neurobs.com); EEG: Vision Recorder (www.BrainProducts.com)
Data analysis	BrainVoyager v21 (www.brainvoyager.com), MATLAB 2018a (www.mathworks.com), FieldTrip toolbox (http://www.fieldtriptoolbox.org), Vision Analyzer 2.1 (www.BrainProducts.com)

For manuscripts utilizing custom algorithms or software that are central to the research but not yet described in published literature, software must be made available to editors/reviewers. We strongly encourage code deposition in a community repository (e.g. GitHub). See the Nature Research guidelines for submitting code & software for further information.

Data

Policy information about availability of data

All manuscripts must include a data availability statement. This statement should provide the following information, where applicable:

- Accession codes, unique identifiers, or web links for publicly available datasets
- A list of figures that have associated raw data
- A description of any restrictions on data availability

The datasets generated and/or analysed during the current study are available from the corresponding author on request.

Field-specific reporting

Please select the one below that is the best fit for your research. If you are not sure, read the appropriate sections before making your selection.

☒ Life sciences ☐ Behavioural & social sciences ☐ Ecological, evolutionary & environmental sciences

Life sciences study design

All studies must disclose on these points even when the disclosure is negative.

Sample size	The sample size of n = 4 was based on theoretical and methodological considerations. This study focuses on the benefits of concurrent TMS-EEG-fMRI measurements for comprehensive neural assessments within individuals. This approach fits potential future applications of this novel method (improved diagnostics and assessment of therapeutic effects, establishing personalized treatment protocols for neurorehabilitation, etc.), for which generalization to the population level is less relevant.
Data exclusions	Participants were excluded based on the exclusion criteria as mentioned in the manuscript.
Replication	Results may not generalize to participants with intrinsic low motor network reactivity (i.e., low and confined network activations during Motor Execution, suggesting weaker connectivity, confirmed by low TMS-evoked network responses), due to overall low motor responses leading to limitations in ROI identification and detection of TMS-evoked activity. Future studies stimulating PreMotor Cortex could benefit from preselecting participants for TMS-EEG-fMRI based on adequate motor network reactivity during Motor Execution.
Randomization	NA (within-subject design).
Blinding	NA (within-subject design). Note that both experimenters as well as participants were blinded to the independent experimental variable (EEG alpha power)

Reporting for specific materials, systems and methods

We require information from authors about some types of materials, experimental systems and methods used in many studies. Here, indicate whether each material, system or method listed is relevant to your study. If you are not sure if a list item applies to your research, read the appropriate section before selecting a response.

Materials & experimental systems

n/a	Involved in the study	
☒	☐	Antibodies
☒	☐	Eukaryotic cell lines
☒	☐	Palaeontology
☒	☐	Animals and other organisms
☐	☒	Human research participants
☒	☐	Clinical data

Methods

n/a	Involved in the study	
☒	☐	ChIP-seq
☒	☐	Flow cytometry
☐	☒	MRI-based neuroimaging

Human research participants

Policy information about studies involving human research participants

Population characteristics	Four healthy, right-handed volunteers (2 males; mean age 26.3 ± 2.7 years) participated in two fMRI sessions (main study and localizer study). None of the subjects met any of the fMRI and TMS exclusion criteria and all of them had previously participated in TMS, EEG, and fMRI experiments.
Recruitment	Voluntary recruitment of participants experienced with multimodal imaging. Note that any voluntary recruitment bias is unlikely to bias results, as we measured basic, physiological responses (BOLD responses) to unpredictable stimulation (TMS) events during resting state.
Ethics oversight	Medical ethical committee of Maastricht University Medical Centre.

Note that full information on the approval of the study protocol must also be provided in the manuscript.

Magnetic resonance imaging

Experimental design

Design type	event-related design in main study (TMS events); block design in fMRI localizer study (motor-execution & auditory cues only control task)
Design specifications	Main study:<br>- Neuronavigated TMS stimulation of the human motor system in a concurrent TMS-EEG-fMRI set-up (for methodological details on acquisition and analyses; see Peters et al. 2013; Journal of Neurophysiology).<br>- Events of Interest:<br>1) TMS-evoked fMRI BOLD responses. Event duration: 133 ms (15 Hz TMS pulse triplets). Inter-event interval:

11.25-15.75 s. Number of events: 120-150 events per participant.
2) . Null-events (matched to TMS-events in terms of their number of occurrences and temporal distribution) during which no TMS stimulation was delivered (control analyses).

fMRI localizer study:
- standard motor-execution localizer task (auditory paced finger tapping and auditory only control blocks). Block duration: 4s. Inter-block interval: 12-18 s. Number of blocks: 26 motor execution & 26 auditory only blocks per participant.

Behavioral performance measures

N.A.

Acquisition

Imaging type(s)

functional MRI (BOLD response), structural MRI

Field strength

3 Tesla

Sequence & imaging parameters

MR session 1: MRI localizer study
- echo-planar imaging (EPI); 24 transversal slices; TR/TE = 1500/30 ms; FA = 71°; bandwidth = 2112 Hz/Px; 3.5 mm isotropic nominal resolution
- magnetization-prepared rapid acquisition gradient echo sequence (MPRAGE); 192 sagittal slices; FoV 256×256 mm; TR/TE=2250/2.6 ms; FA=9°; 1.0 mm isotropic nominal resolution.

MR session 2: Concurrent TMS-EEG-fMRI (main study)
- EPI 21 transversal slices; TR/TE = 2250/30 ms; gap = 750 ms; FA = 78°; bandwidth = 2112 Hz/Px; 3.5 mm isotropic nominal; 313 volumes per run
- MPRAGE; 192 sagittal slices; FoV 128×128 mm; TR/TE=2250/2.6 ms; FA=9°; 2.0 mm isotropic nominal resolution

Area of acquisition

cortical and subcortical areas

Diffusion MRI ☐ Used ☒ Not used

Preprocessing

Preprocessing software

BrainVoyager software package v21 (Brain Innovation, Maastricht, the Netherlands)

Normalization

For each run, the voxel's BOLD timecourse was normalized (z-score). No other normalization procedures were needed (participant specific, functionally defined ROIs; neuronavigated TMS-coil position based on participant-specific target ROI coordinates)

Normalization template

N.A.

Noise and artifact removal

Preprocessing of all fMRI data included slice scan time correction, linear trend removal, high-pass filtering, and three-dimensional motion correction. The first two volumes of each run were discarded to remove T1 saturation effects. Functional data were not spatially smoothed, but a temporal high-pass filter was applied to remove frequencies lower than 2 cycles per time course. Gray-white matter borders were extracted, from which individual cortical surface meshes were reconstructed for TMS neuronavigation (see Sack et al., 2009). For statistical analyses (GLM), data were z-normalized and corrected for serial correlations using a second-order autoregressive model.

Volume censoring

N.A.

Statistical modeling & inference

Model type and settings

Main study:
- non-parametric Wilcoxon sign rank test; univariate, permutation testing (null distributions based on 20000 random-label permutations per test); voxel-wise FFX-GLM (to obtain TMS-responsive ROIs).

Localizer study:
- univariate whole brain, voxel-wise FFX-GLM

Effect(s) tested

Main study:
1 TMS-evoked BOLD-responses (beta weight estimates) as a function of EEG alpha power in motor ROIs (main) and non-motor ROIs (control)
2 BOLD-responses related to (control) Null-events, as a function of EEG alpha power in motor ROIs (main) and non-motor ROIs (control)
3 TMS-evoked BOLD-responses as a function of EEG beta power (control analyses to test specificity of effects for the alpha band).
4 relation between trial order and alpha power (control analyses for alpha drifts).
5 EMG responses to TMS-events versus Null-events.

Localizer study:
1 BOLD-response during motor-execution (+ auditory stimulation) compared to only auditory stimulation and resting

baseline.

Specify type of analysis: ☐ Whole brain ☐ ROI-based ☒ Both

Anatomical location(s)	Main study: Functionally localized (motor ROIs) and anatomical (non-Motor ROIs) Regions-Of-Interest (ROIs).<br>No anatomical atlases were used for main analyses (motor ROIs, whole brain), for control analyses (non-Motor ROIs) we used the Brodmann atlas (in Talairach-space) projected on the individual anatomical space of each participant.<br><br>Localizer study: whole brain.
Statistic type for inference<br>(See Eklund et al. 2016)	Voxel-wise
Correction	Voxel-wise using the false discovery rate (FDR) of q=0.05 (Benjamini & Hochberg, 1995)

Models & analysis

n/a	Involved in the study
☐	☒ Functional and/or effective connectivity
☒	☐ Graph analysis
☒	☐ Multivariate modeling or predictive analysis

Functional and/or effective connectivity	fMRI activity (BOLD beta estimates) in remote areas induced by noninvasive brain stimulation (TMS), as assessed by permutation testing. Note that this measured activity indicates the propagation of the focally induced neural activity (by TMS) to structurally and functionally connected areas and thus reflects actual effective connectivity, rather than a construed connectivity measure (derived from connectivity analyses) .
